# Bioactive Insecticides from Chemometric Diverse Ant-Associated Symbionts *Streptomyces novaecaesareae* and *Streptomyces nojiriensis* against the Fall Armyworm Larvae

**DOI:** 10.3390/insects15090707

**Published:** 2024-09-17

**Authors:** Cecília Beatriz Nascimento Lima, Mariana Montini Joly, Luiz Alberto Beraldo Moraes, Fernando Luís Cônsoli

**Affiliations:** 1 Insect Interactions Laboratory, Luiz de Queiroz College of Agriculture, University of São Paulo, Piracicaba 13418-900, SP, Brazil; 2 Chemistry Department, Faculty of Philosophy, Sciences and Letters, University of São Paulo, Ribeirão Preto 14040-900, SP, Brazil

**Keywords:** microbial intraspecific diversity, natural compounds, sustainable pest management, symbiont-derived insecticides

## Abstract

**Simple Summary:**

The within-species diversity of *Streptomyces nojiriensis* and *Streptomyces novaecaesareae* in producing bioactive compounds against *Spodoptera frugiperda* larvae is demonstrated, leading to the identification of valinomycin and naphtomycin from *S. novaecaesareae* as the bioactive molecules. The potential use of these molecules as alternative strategies for the sustainable management of *Spodoptera frugiperda* larvae is discussed.

**Abstract:**

The *Streptomyces* genus has long been recognized as a prolific and valuable source of diverse secondary metabolites. These metabolites contribute significantly to the intricate chemical diversity exhibited by *Streptomyces*, making them an indispensable reservoir for drug discovery, agricultural applications, and industrial processes. Exploiting the potential of these natural compounds holds the promise of ushering in a new era in insect pest management, reducing reliance on synthetic chemicals and fostering ecologically sustainable solutions. This study dives into the realm of chemo diversity within isolates of *Streptomyces nojiriensis* and *Streptomyces novaecaesareae*, with a specific focus on the production of insecticidal compounds. We explored chromatographic techniques for the identification and isolation of insecticidal compounds, and two bioactive compounds were identified in extracts of *S. novaecaesareae*. Valinomycin was identified from hexanic extracts of strain Asp59, while naphthomycin from ethyl acetate extracts of strain Asp58. These compounds showed insecticidal activity against first instars of *Spodoptera frugiperda* (Asp59: LC_50_ = 10.82 µg/µL, LC_90_ = 26.25 µg/µL; Asp58: LC_50_ = 15.05 µg/µL, LC_90_ = 38.84 µg/µL). Notably, this is the first report of naphthomycin as an insecticidal compound. The present study suggests that valinomycin and naphthomycin may be a novel biological source for the control of *Spodoptera frugiperda* in early stages.

## 1. Introduction

The fall armyworm (FAW) *Spodoptera frugiperda* (J.E. Smith) (*Lepidoptera: Noctuidae*) has become a worldwide pest after its spread from the New World to Africa in 2016 [1], and then through Asia [2,3] and Oceania [4]. The FAW has also reached Europe, where it has been recently reported in Greece [5], Portugal [6], and Romania [7]. Risk assessment models have projected the Mediterranean coastal areas of the Southern Europe are suitable for the fall armyworm establishment, with an expected collection of 5 to 139 moths per trap per week and up to four generations per year. Areas in Central Europe up to the 48th parallel north are at risk of occurrence of host transient populations of this pest [8]. Chemical control remains the most widely used method, relying on synthetic insecticides such as pyrethroids and organophosphates. However, resistance evolution in FAW populations significantly undermines the efficacy of these chemicals, necessitating higher doses or alternative compounds, which raises environmental and health concerns [9]. Resistance evolution has been shown to occur even against several events available in genetically modified (GM) crops expressing *Bacillus thuringiensis* (Bt) toxins, rapidly reducing the lifespan of this technology [10]. The use of biological control strategies with parasitoids, predators, and entomopathogens is often attempted but with very little efficacy due to the need for specific environmental conditions and the slow action of biocontrol agents [11]. Cultural practices, such as crop rotation and intercropping, provide non-chemical options that can suppress pest populations; however, their effectiveness is contingent on proper implementation and local agronomic conditions [12]. Therefore, the invasive abilities of the FAW and its destructive potential [13,14] and high capacity of adaptation to several chemical and Bt-based strategies of pest management pose serious threats to food security [15,16,17] and to the development of agriculture practices to meet the goals for sustainable development in the Agenda 2030 [18]. Such threats are leading to the search for alternative strategies for sustainable FAW control [19]. *Streptomyces* is a filamentous, spore-forming, Gram-positive bacterium belonging to the *Actinomycetota* phylum [20,21]. It can be found in diverse environments, including symbiotic relationships with invertebrates [22] and plants [23], as well as in extreme and underexplored habitats in terrestrial and marine regions [24,25]. A remarkable feature of *Streptomyces* is the presence of multiple biosynthetic gene clusters (BGCs) within each genome. These gene clusters are responsible for producing numerous bioactive compounds that hold great potential for biotechnological exploitation. The biotechnological potential of *Streptomyces* as a source of bioactive molecules with antimicrobial activity has long been demonstrated [26]. Moreover, the biological and chemical diversity in this highly active bacterial genus has expanded the potential of *Streptomyces*-produced compounds to several biotechnological and industrial applications, such as antimicrobial, antiviral, cytotoxic, antitumor, antihypertensive, immunosuppressive, antioxidative, plant-growth-promoting, and herbal agents [27,28,29,30,31], including their use in agriculture for insect pest control [32,33,34]. For example, the novel polyketide antibiotic *streptomycin C* has demonstrated potent activity against multi-drug-resistant bacterial strains, offering a promising solution to the growing issue of antibiotic resistance [35]. Additionally, *Streptomyces* strains have been found to produce unique antitumor agents, such as *streptosetin*, which exhibits cytotoxic activity against various cancer cell lines through apoptosis induction and cell cycle arrest [36]. Another notable discovery is the identification of new immunosuppressive agents like *streptolide*, which modulates the immune response by targeting specific signaling pathways, presenting a potential therapeutic avenue for autoimmune diseases [37]. These findings demonstrate the immense biochemical diversity of *Streptomyces* and highlight their critical role in the discovery and development of new bioactive molecules. The exploitation of the full chemical diversity of microbial compounds with potential biotechnological application is often guided by the one strain many compounds (OSMAC) approach or the use of elicitors and the co-cultivation techniques to activate silent genes. The elicitation of silent genes has proven effective in activating hidden metabolic pathways and in the production and identification of new active metabolites [34,38,39,40].

However, despite the differential activation of gene clusters in response to biotic and abiotic stressors supporting the one strain many compounds strategy to search for new active secondary metabolites, there is an increasing body of evidence demonstrating significant intraspecific variation in actinobacteria. In *Streptomyces*, such intraspecific variation in metabolite production has been observed even among strains that share the same core of specialized metabolite biosynthetic gene clusters, as strain-specific gene clusters can still occur [41,42]. Intraspecific variations are more commonly observed among *Streptomyces* strains originating from different locations [42,43]. These genomic differences and the strain-level chemical diversity have been suggested as intraspecific adaptive traits of *Streptomyces* strains to different stressors associated with their ecological niches [42].

In an early study [44], 106 bacterial morphotypes were isolated from the cuticle surface of worker ants of *Acromyrmex coronatus* (Fabricius) (*Hymenoptera*, *Formicidae*) using several culture media (ISP4, SM3, TSA, ISP2, and chitin). Sixty-five of these isolates belonging to *Actinomycetota* were further examined for their biological and chemical diversity, resulting in the identification of an impressive chemical multiplicity among strains belonging to several different phylotypes studied [38]. Among the isolates, strains putatively identified as *Streptomyces nojiriensis* and *Streptomyces novaecaesareae* were categorized into different groups using Principal Component Analysis based on their chemometric profiles. Furthermore, the biological activity of these strains also varied, indicating that they produce a diverse set of insecticide-active compounds [44]. The objective of this study was to conduct further investigations into the diversity of compounds produced by a subset of *S. nojiriensis* and *S. novaecaesareae* strains and identify the active compounds produced.

We anticipate that our investigation into the potential of strain-level chemical diversity to contribute with the discovery of *Streptomyces*-produced insecticides will enhance our chances to identify new compounds or new activities for known compounds. This contribution can play a role in implementing low-risk, environmentally friendly strategies for pest control, aligning agricultural food production with the United Nation’s goals for sustainable development.

## 2. Materials and Methods

### 2.1. Bacterial Isolates

Several isolates from two phylotypes obtained from the cuticle surface of the leafcutter ant *Acromyrmex coronatus* and putatively identified as *S. nojiriensis* (isolates IILAsp39, IILAsp89, IILAsp90, IILAsp93, IILAsp96, and IILAsp101b) and *S. novaecaesareae* (IILAsp45, IILAsp49, IILAsp58, IILAsp59, and IILAsp85b-1) through heuristic comparisons of nearly complete sequences of the 16S rRNA gene [44] were selected for this study.

Aliquots of the selected isolates were retrieved from 20% glycerol stocks stored at −80 °C and streaked on ISP2 and ISP4-agar plates. The plates were incubated at 28 °C for 7 days or until colony growth was visible. Subsequently, the obtained colonies were cultured to confirm their identification through the amplification and sequencing of the 16S ribosomal RNA gene. Colonies were picked from the plates and cultured in 5 mL of ISP2 medium at 28 °C for 7 days under constant agitation (120 rpm). Following this, cells were pelleted by centrifugation (2000× *g* × 5 min) and used for genomic DNA extraction using the phenol–chloroform method. Aliquots of 1000 µL from the culture were added to 2 mL safe-lock tubes with 500 µL of Tris-HCl (pH 8.0)-saturated phenol at room temperature. After centrifugation at 7500× *g* for 5 min, the aqueous phase was collected and subsequently extracted with 500 µL of Tris-HCl (pH 8.0)-saturated phenol–chloroform (1:1) and two times with chloroform. Finally, the aqueous phase was precipitated with a 1:1 volume of 100% isopropanol for 60 min at −20 °C. After centrifugation at 11,000× *g* for 10 min, the supernatant was discarded, and the pellet was washed twice with 1000 µL of 70% ethanol After centrifugation at 11,000× *g* for 10 min, the supernatant was discarded and the pellet dried and resuspended with 20 µL of ultrapure water. The quality and integrity of DNA were assessed through 0.8% (*w*/*v*) agarose gel electrophoresis containing 0.5 µg∙mL^−1^ ethidium bromide at 70 V for 1 h in TAE buffer (40 mM Tris-acetate, 1 mM EDTA, pH 7.2) and standard A260/280 ratio obtained in spectrophotometric analysis. 

The DNA samples underwent partial PCR amplification of the 16S rRNA gene. PCR reactions consisted of 10–20 ng of genomic DNA, 0.32 µM of each universal primer (8F: AGAGTTTGATGCCTCAG; 1491R: GGTTACCTTGTTACGACTT), 1× enzyme buffer, 1.5 mM MgCl_2_, 0.2 mM of each dNTP, and 0.625 U of Taq polymerase (Promega, Madison, WA, USA), in a reaction with a final volume of 25 µL. The amplifications were carried out in a thermocycler programmed at 95 °C for 4 min (1 cycle); 95 °C for 1 min, 55 °C for 1 min, and 72 °C for 2 min (35 cycles); and 72 °C for 10 min (1 cycle). The amplicons obtained were revealed by standard DNA electrophoresis on a 1% agarose gel slab containing 0.5 µg∙mL^−1^ ethidium bromide under constant voltage (100 V) in TAE buffer [45].

The obtained amplicons were sequenced using Sanger´s in one of the facilities of the University of São Paulo. The sequences obtained were analyzed and edited using Finch TV v1.4.0 (Geospiza Inc., Seattle, WA, USA). The merging of forward and reverse reads resulted in a single ~1350 bp-long sequence. The merged 16S rDNA sequences were compared to the previous 16S rDNA sequences made available by Martinez et al. (2019) [44] for putative identification and confirmation of the selected isolates.

### 2.2. Determination of the Chemical Profile of Crude Extracts

#### 2.2.1. Crude Extract Preparation

The selected isolates were cultured in 1.5 L of ISP4 or ISP2 medium and incubated for 7 days at 28 ± 1 °C to ensure optimal growth conditions. Incubation was performed under continuous shaking at 120 rpm to maintain aeration and homogeneity. Following the incubation period, the bacterial cultures were subjected to centrifugation at 3000× *g* × 10 min to separate the bacterial cells from the culture medium. The supernatant, which contains extracellular metabolites, was then carefully filtered to remove any remaining particulate matter. Each isolate´s supernatants were subjected to individual 1:1 liquid–liquid extractions with hexane and with ethyl acetate using standard liquid extraction procedures [44]. These two extraction methods were chosen due to the complementary extraction power of hexane and ethyl acetate, aiming to recover the broadest range of compounds possibly produced by each isolate. The obtained organic extracts were concentrated in a rotary evaporator (40 °C, 600 mbar of vacuum). The dried samples were weighted, stored in amber vials at −20 °C, and further diluted as required.

##### LC-MS Analyses of Crude Extracts

The chemical profile of all crude extracts was obtained by chromatographic analyses performed in a Xevo™ TQ-S (Waters Corporation, Milford, MA, USA) Mass Spectrometer coupled with an Acquity™ Ultra High-Performance Liquid Chromatograph (UPLC™, Waters Corporation, Milford, MA, USA) equipped with a BEH C18 (21 × 100 mm, 1.7 μm) column, a diode array detector, and an auto-sampler. The mass spectrometer operated with electrospray ionization in the positive mode (ESI+). Gradient elution started with 40% phase B, increased linearly to 99% within 5 min, and was maintained in 99% phase B for 2 min. Mobile phase A consisted of 0.1% formic acid in water, and mobile phase B consisted of 0.1% formic acid in acetonitrile. The flow rate was 400 μL∙min^−1^, and the injection volume was 10 μL. Crude extracts were diluted to a final concentration of 300 µg∙mL^−1^. Data analysis was performed using MassLynx Software version 4.1 and Progenesis QI software v3.0 (Waters Corporation, Milford, MA, USA). 

The data acquired in control samples were used to filter out compounds that were associated with the metabolism of bacteria. The filtered data obtained were subjected to metabolomics data analysis using the resources implemented in the MetaboAnalyst 5.0 platform (https://www.metaboanalyst.ca/, accessed on 15 November 2023). The data were normalized with the method “by sum” to adjust the abundance of each compound to a fixed value and reduce variations among samples and compared using a one-way ANOVA and post hoc statistics to identify differences among isolates for each compound produced. 

The data obtained were also subjected to Principal Component Analysis (PCA) to examine the correlation between the major compounds and the yield of isolates across different extraction methods. This analysis facilitated the classification of similar isolates based on yield and the major compounds identified. Moreover, PCA allowed for a clear visualization of the distinct profiles among isolates, considering the organic solvents used for compound isolation. The dataset consisted of samples obtained from the two extraction methods. The variables considered were the average of each identified compound and the principal components representing the chemical composition of each strain within each *Streptomyces* species. 

### 2.3. Bioassays, Isolation, and Identification of Bioactive Insecticide Compounds

#### 2.3.1. Crude Extract Insecticide Activity

The insecticidal activity of hexane and ethyl acetate extracts obtained from the fermentation broth of all investigated isolates was tested against the first instars of *S. frugiperda*. Larvae of *S. frugiperda* were obtained from a susceptible reference strain that had been maintained under laboratory conditions without insecticide selection pressure for years on a bean-based artificial diet [46]. Feeding assays were conducted in sterile 24-well plates, with each well filled with 1.25 mL of bean-based artificial diet. After the diet cooled, 20 μL crude extract in acetone (25 μg∙μL^−1^) was applied to the diet surface, resulting in 260 μg∙cm^2^ of crude extract in each well. Plates were kept open in a laminar flow hood for 1 h to allow for complete evaporation of the solvent. Then, each well was inoculated with 10 larvae (0–24 h old) for larval exposure to the surface-treated artificial diet, with a total of 240 larvae/treatment. To ensure the reliability of the results, the assay was conducted with 24 repetitions and repeated three times. All plates were sealed with Parafilm^®^ containing pinholes for ventilation. The effects of the solvent were assayed by adding 20 μL of acetone to the artificial diet under the same conditions used in the bioassay (control treatment). Samples were maintained under controlled conditions (25 ± 2 °C; 60 ± 10% RH; 14 h photophase). Larval mortality was evaluated daily for 5 days, considering larvae as dead when unresponsive to touch. 

The data were analyzed using Abbott´s formula to correct for natural mortality observed in control group. Following this correction, an Analysis of Variance (ANOVA) was conducted to determine if there were significant differences in mortality rates between the treatment groups. When ANOVA indicated significant differences, the Tukey Honet Significant Difference (HSD) test was performed as a *post hoc* analysis using the *glht* function from multcomp package with *p*-value adjustment to identify which specific treatment differed from each other. All analyses were performed using the statistical software RStudio ^®^ version 2.15.1.

#### 2.3.2. Isolation of Bioactive Insecticidal Compounds

The bioactive insecticidal compounds were isolated from crude extracts using bio-guided assays. The insecticidal activity of the obtained fractions was tested following the previously described bioassay. Two extracts were selected based on their insecticidal activity for the isolation of insecticidal compounds: the ethyl acetate extract of isolate Asp58 and the hexane extract of isolate Asp59.

The ethyl acetate crude extract of Asp58 was subjected to methanol: hexane (1:1) liquid–liquid extraction to separate partitions with different chemical affinities. Each gram of the dried ethyl acetate crude extract of Asp58 was first solubilized in 100 mL of methanol, and then, 100 mL of hexane was added for sample partitioning. The procedure was repeated three times. The methanol fraction AE58M recovered 52% of the original extract, and the hexanic fraction AE58H 48%.

The hexane extract of the isolate Asp59 was subjected to fractionation using a silica column chromatography (glass column 40 cm high × 2 cm in diameter packed with silica gel in hexane in a 1 g:4 mL ratio). The column was initially conditioned with hexane. Then, 1 g of the Asp59 hexanic extract was solubilized in hexane and applied to the column. A set of organic solvent combinations was applied to separate the bioactive compounds. Thus, 20 mL of each of the following combinations was applied successively: 100% hexane; 95% hexane: 5% ethyl acetate; 90% hexane: 10% ethyl acetate; 80% hexane: 10% ethyl acetate; 70% hexane: 30% ethyl acetate; 60% hexane: 40% ethyl acetate; 50% hexane: 50% ethyl acetate; 40% hexane: 60% ethyl acetate; 30% hexane: 70% ethyl acetate; 20% hexane: 80% ethyl acetate; 10% hexane: 90% ethyl acetate and 100% ethyl acetate, resulting in 12 fractions. The 12 fractions were subjected to thin layer chromatography (TLC) and observed at wavelengths of 254 nm and 365 nm. Fractions were grouped by chemical similarity into six samples: H59A (1.6% yield), H59B (37.3%), H59C (43.6%), H59D (7.9%), H59E (0.8%), and H69F (8.7%). 

After bioassaying these samples, a mass of 300 mg of the H59B fraction was subjected to analytical and semi-preparative chromatographic analyses in a Shimadzu HPLC system equipped with a CBM-20A controller, two LC-6AD pumps, a DGU-20A5 degasser, SPD-20 UV-Vis’s detector, and an automatic sample collector. The column used in the analytical system was a Shimadzu ODS H (250 × 4.6 mm; 5 μm) (Shimadzu Corporation, Kyoto, Japan). The column used in the semi-preparative system was an Agilent Eclipse XDB (250 × 9.4 mm; 5 μm) (Agilent Technologies, Santa Clara, USA). The analytical analysis used an elution gradient starting with 90% water + 0.1% formic acid (A) and 10% methanol + 0.1% formic acid (B), with the gradient of A varying from 90% to 70% in the interval from 0 to 10 min and 70% to 5% in the interval from 10 to 50 min. The flow rate used was 1.0 mL∙min^−1^, and the injection volume was 20 μL. The wavelengths studied were 370 and 305 nm. The same method was transferred to a semi-preparative scale using the same chromatographic system but with a flow rate of 13.0 mL.min^−1^ and an injection volume of 1000 μL. After the preparative chromatographic isolation, 48 fractions were obtained. Thin-layer chromatography was performed to group fractions that had a similar chemical profile, thus optimizing the bioassays. The fractions 0–12 (called group A), 13–22 (group B), 23–32 (group C), 32–37 (group D), and 38 to 48 (group E) were tested.

### 2.4. Determination of Concentration—Response Curves of Isolated Insecticidal Bioactive Compounds

After isolating the active compounds, a concentration–response curve was constructed to estimate the lethal concentrations necessary to kill 50 (LC_50_) and 90% (LC_90_) of the *S. frugiperda* population. The range of concentrations used was determined using Finney’s formula after conducting preliminary tests to establish baseline concentrations (i.e., natural mortality as observed in the control treatment and 99% mortality of *S. frugiperda* larvae). The concentrations tested ranged from 5 μg∙μL^−1^ to 30 μg∙μL^−1^, with a total of 11 concentrations tested. The bioassays followed the same experimental procedure as described earlier in the feeding assays.

## 3. Results

### 3.1. Intraspecies Variation in the Chemical Profile among Isolates of S. nojiriensis and S. novaecesareae 

#### 3.1.1. Chemical Diversity of *S. nojiriensis* Isolates

**Figure 1:**
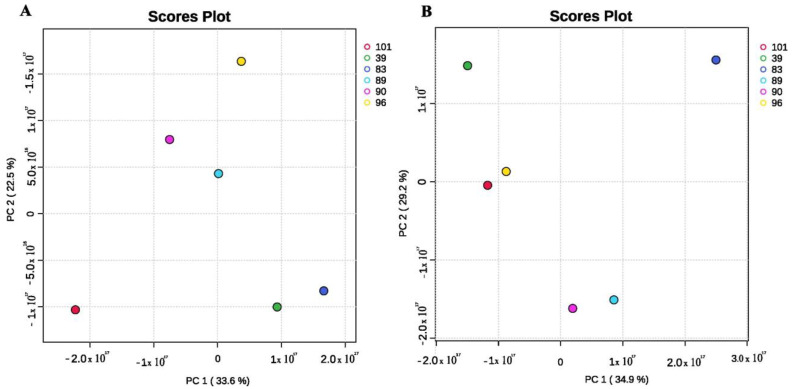
Score plots of different strains of *S. nojiriensis* based on the compounds available in their ethyl acetate (**A**) and hexane (**B**) crude extracts, demonstrating the intraspecies variation in metabolite profiles.

A Principal Component Analysis (PCA) of the chemical diversity identified in the ethyl acetate extracts of *S. nojiriensis* strains revealed four distinct clusters. Strains Asp39 and Asp83 resolved in the same quadrant, while strains Asp90, Asp96, and Asp101 resolved each in an individual quadrant. Asp89 did not resolve in a clear quadrant but had Asp90 as the closest strain (Figure 1). In contrast, PCA based on the diversity of metabolites in hexanic extracts of *S. nojiriensis* yielded different grouping patterns compared to the ethyl acetate extracts. Isolates Asp83 and Asp101 were individually located in opposite quadrants. Asp39 and Asp96 grouped together, but Asp96 was closer to Asp101 than to Asp39. Asp89 and Asp90 grouped close together in the same quadrant (Figure 1).

The Principal Component Analysis (PCA) of the chemical diversity identified in the ethyl acetate extracts of *S. nojiriensis* strains grouped them into four quadrants. Strains Asp39 and Asp83 resolved in the same quadrant, while strains Asp90, Asp96, and Asp101 resolved each in an individual quadrant. Asp89 did not resolve in a clear quadrant but had Asp90 as the closest strain (Figure 1). In contrast, a PCA based on the diversity of metabolites in hexanic extracts of *S. nojiriensis* yielded different grouping patterns compared to the ethyl acetate extracts. Isolates Asp83 and Asp101 were individually located in opposite quadrants. Asp39 and Asp96 grouped together, but Asp96 was closer to Asp101 than to Asp39. Asp89 and Asp90 grouped close together in the same quadrant (Figure 1).

**Figure 2:**
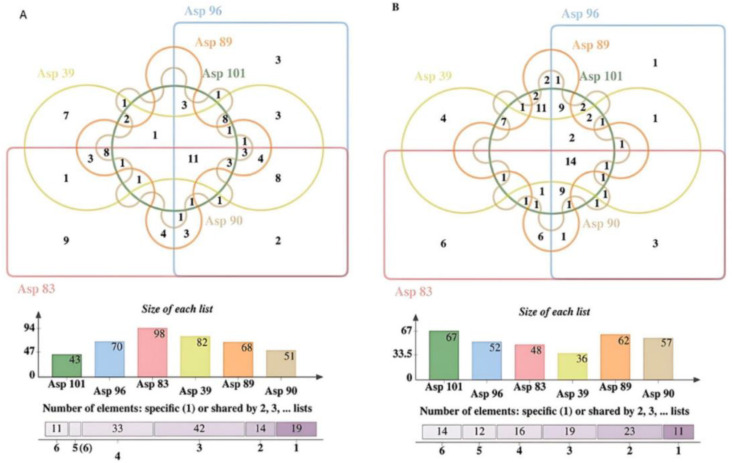
Venn diagrams representing the overlap and numbers of compounds in ethyl acetate (**A**) or hexane extraction (**B**) crude extracts of different isolates (Asp 101, Asp96, Asp 83, Asp39, Asp89 and Asp90) of *S. nojiriensis* based on LC-MS/MS identification. Please refer to Supplementary Table S1 for the full list of compounds identified.

**Figure 3:**
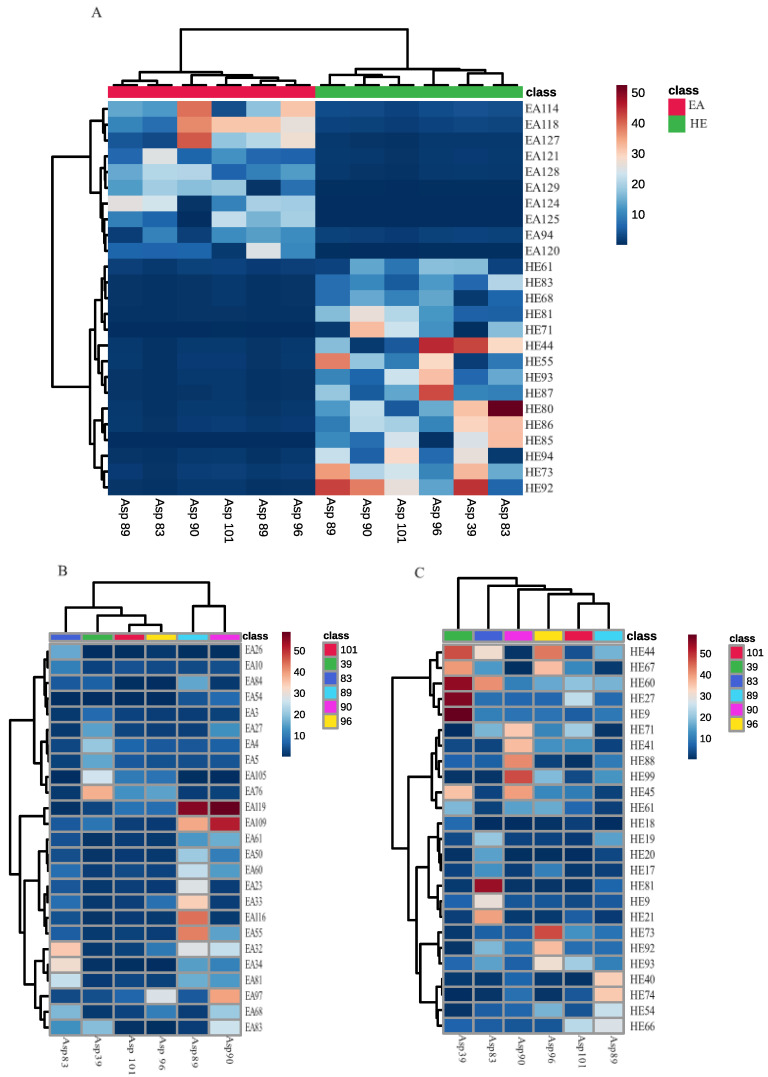
Metabolite abundance and occurrence in ethyl acetate (EA) and hexane (HE) extracts obtained from different strains of *S. nojiriensis*, based on LC-MS metabolomics analysis. (**A**) Compounds common to all strains of *S. nojiriensis*; (**B**) Compounds that are not produced by all strains and that are differentially abundant in ethyl acetate extracts; (**C**) Compounds that are not produced by all strains and that are differentially abundant in hexane extracts. Compound abundance in each sample is represented in color scale from red (highest abundance) to blue (lowest abundance). Please refer to Supplementary Table S1 for the full list of compounds identified and their complete chemical names.

The ethyl acetate and hexane extracts obtained from crude fermentations of *S***.** *nojiriensis* strains exhibited high intraspecific qualitative and quantitative differences (Figure 2 and Figure 3A, Supplementary Table S1). The use of these organic solvents for metabolite extraction produced by *S. nojiriensis* strains resulted in an additive yield of compounds, with the ethyl acetate extract containing 129 compounds and the hexanic extract containing 97 compounds. Overall, strain Asp83 of *S. nojiriensis* was the most active biologically with a total of 146 compounds identified, while 108 compounds were identified in extracts from Asp90 (Figure 2, Supplementary Table S1). The most diverse ethyl acetate extract was that of Asp83 (98) and the hexanic extract was that of Asp101 (67). A total of 30 compounds (19 EA; 11 H) were unique. Asp83 produced most of the unique compounds (9) identified in ethyl acetate extracts and in hexanic extracts (6) (Figure 2, Supplementary Table S1). Only 11 out of the 129 compounds identified in ethyl acetate extracts, and 14 out of the 97 compounds identified in hexanic extracts were common to all strains of *S. nojiriensis* analyzed (Figure 2, Supplementary Table S1). These findings highlight the importance of the extraction method in demonstrating the differential metabolite production capacity among strains of a single phylotype, even when derived from the same environmental condition. (Supplementary Table S1).

The abundance of the putatively identified compounds shared by all *S. nojiriensis* strains in ethyl acetate (EA) and hexane (HE) extracts was strain-dependent (Figure 3A, Supplementary Table S1). But several other compounds were produced by a single strain of only a portion of them in both EA and HE extracts, and these compounds also differed in their abundance when compared in between the strains that produced them (Figure 3B,C, Supplementary Table S1). Asp90 ethyl acetate extract contained the highest abundance (EA97, EA109 and EA117) of Ile Gln Phe Arg, methyl oleoylethanolamide, and methyl 4-[[1-(4-fluorophenyl) sulfonylpyrrolidine-2-carbonyl] amino] benzoate (Figure 3B, Supplementary Table S1). Asp39 extract was characterized by having the highest abundance of N-[1-(2-methoxyethyl)-2-oxo-3,4-dihydroquinolin-6-yl] butanamide (EA76) (Figure 3B, Supplementary Table S1). Ethyl acetate extracts of isolate Asp83 had the highest abundance of 5-[(3-phenyl-1,2,4-oxadiazol-5-yl)methyl]-2-thiophen-2-ylpyrazolo [1,5-d][1,2,4]triazin-4-one (EA32), and [10,13-dimethyl-17-(6-methylheptan-2-yl)-2,3,4,7,8,9,11,12,14,15,16,17-dodecahydro-1H-cyclopenta [a] phenanthren-3-yl] 3-(3,4-dimethyl-5-pentylfuran-2-yl)propanoate (EA34) (Figure 3A, Supplementary Table S1).

Only 14 out of 97 compounds identified in HE of isolates of *S. nojiriensis* were shared by all isolates, and abundance of shared compounds among *S. nojiriensis* strains was highly variable (Figure 2B). 2-(N-ethyl-perfluorooctane-sulfanamido) acetic acid (HE44) was among the compounds of highest abundance in three out of six strains studied, Asp96, Asp83, Asp39 (Figure 3C, Supplementary Table S1). HE44, N-[(2-fluorophenyl)methyl]-1-[2-(1-methylpyrrolo [2,3-b]pyridin-3-yl)ethyl]piperidine-4-carboxamide (HE67), methyl_4-[[1-(4-fluorophenyl)sulfonylpyrrolidine-2-carbonyl]amino]benzoate (HE60), (4aR,8aR)-1-methyl-2-oxo-N-[(4-propan-2-ylphenyl)methyl]-4,5,6,7,8,8a-hexahydro-3H-quinoline-4a-carboxamide (HE27), and Ala-His-Ala (HE9) are the compounds with highest abundance in Asp39 strain. Asp39 produced the largest number (6) of shared compounds in high abundance, while only seven of the shared compounds were produced in high abundance by isolates Asp 83, Asp90, and Asp96 (Figure 3A, Supplementary Table S1).

#### 3.1.2. Chemical Diversity of *S. novaecaesareae* Isolates

**Figure 4:**
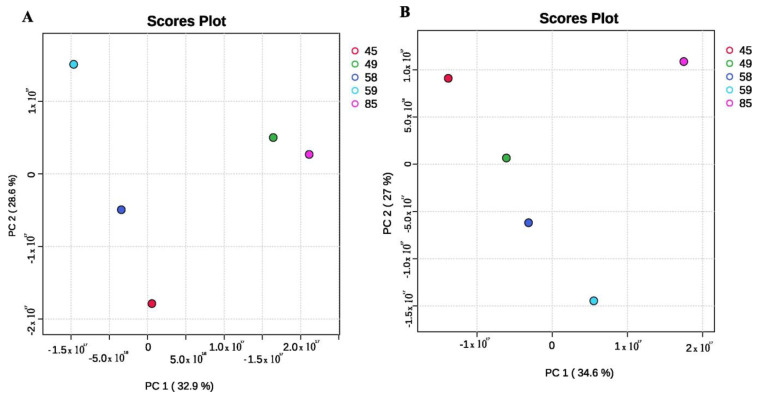
Score plots of different strains of *S. novacaesaereae* based on the compounds available in their ethyl acetate (**A**) and hexane (**B**) crude extracts, demonstrating the intraspecies variation in metabolite profiles.

A Principal Component Analysis (PCA) of the chemical diversity identified in the ethyl acetate extracts of *S. novaecaesareae* strains grouped them into four quadrants. Strains Asp45 and Asp58 formed a group, while Asp59, Asp85, and Asp49 resolved isolated from each other and from the Asp45-Asp58 group (Figure 4). But a PCA based on the diversity of metabolites in hexane extracts of *S. novaecaesareae* yielded different grouping patterns compared to the ethyl acetate extracts. Isolates Asp45 and Asp49 formed a group, while Asp58, Asp59, and Asp85 resolved isolated in separated quadrants (Figure 4).

**Figure 5:**
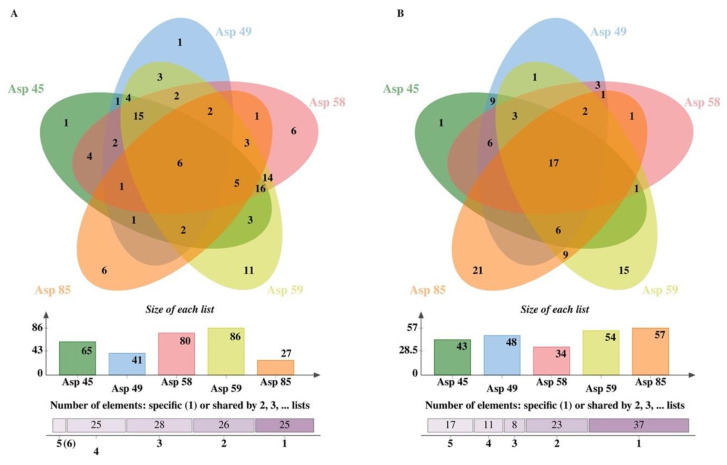
Venn diagrams representing the overlap and number of compounds in ethyl acetate (**A**) or hexane (**B**) crude extracts of different isolates (Asp45, Asp49, Asp58, Asp59 and Asp85) of *S. novaecaesareae* based on HPLC-MS/MS identification. Please refer to Supplementary Table S1 for the full list of compounds identified.

The ethyl acetate and hexane extracts obtained from crude fermentations of *S. novaecaesareae* strains exhibited high intraspecific qualitative and quantitative differences (Figure 5, Supplementary Table S1). The use of these organic solvents for metabolite extraction produced by *S. novaecaesareae* strains resulted in an additive yield of compounds, with the ethyl acetate extract containing 109 compounds and the hexane extract 88 compounds. Overall, strain Asp59 of *S. novaecaesareae* was the most biologically active with a total of 86 compounds identified, while 80 compounds were identified in extracts from Asp58 (Figure 5). But the most diverse ethyl acetate extract was that of Asp59 (96) and the hexane extract was that of Asp85 (57). A total of 31 compounds (19 EA; 12 H) were unique. Asp59 produced most of the unique compounds (11) identified in ethyl acetate extracts and in hexane extracts (6) (Figure 5, Supplementary Table S1). Only 6 out of the 109 compounds identified in ethyl acetate extracts, and only 17 out of the 88 compounds identified in hexane extracts were common to all strains of *S. novaecaesareae* analyzed (Figure 5, Supplementary Table S1). This finding also highlights the importance of the extraction method in demonstrating the differential metabolite production capacity among strains of a single phylotype, even when derived from the same environmental source (Figure 5).

**Figure 6:**
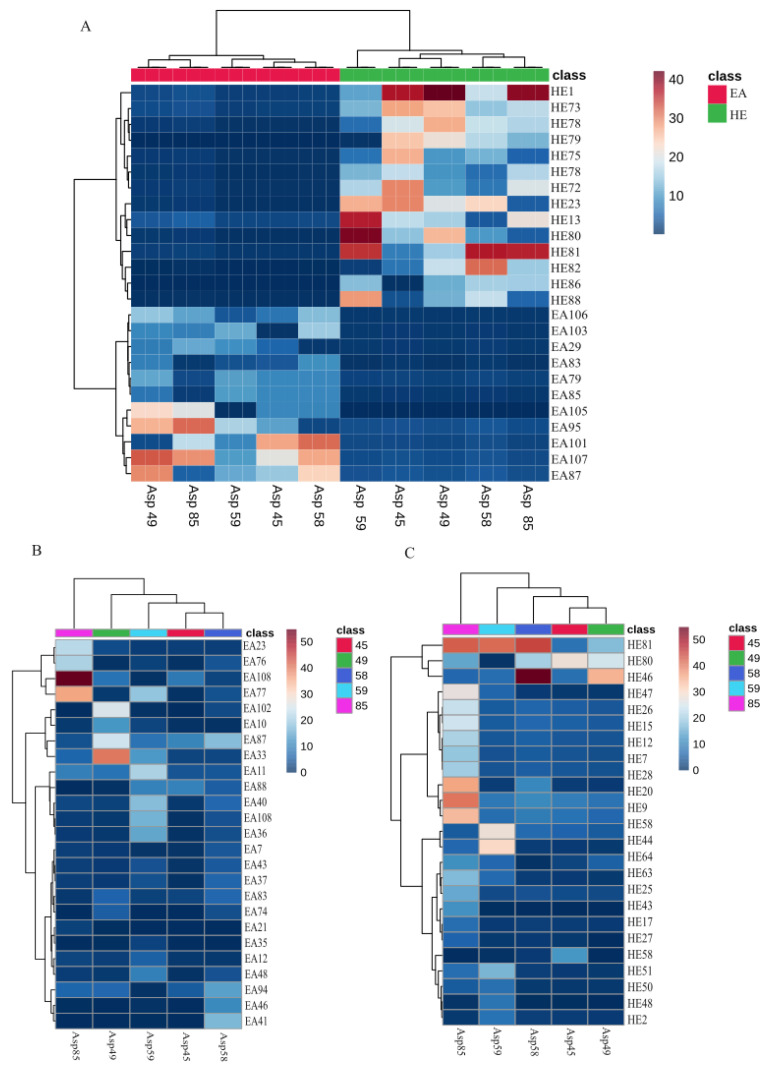
Metabolite abundance and occurrence in ethyl acetate (EA) and hexane (HE) extracts obtained from different strains of *S. novaecaesareae*, based on LC-MS metabolomics analysis. (**A**) Compounds common to all strains of *S. novaecaesareae*; (**B**) Compounds that are not produced by all strains and that are differentially abundant in ethyl acetate extracts; (**C**) Compounds that are not produced by all strains and that are differentially abundant in hexane extracts. Compound abundance in each sample is represented in color scale from red (highest abundance) to blue (lowest abundance). Please refer to Supplementary Table S1 for the full list of compounds identified and their complete chemical names.

The abundance of the putatively identified compounds shared in EA (6) and HE (17) extracts from all *S. novaecaesareae* strains was strain-dependent (Figure 5A, Supplementary Table S1). Several other compounds that were produced only by a portion of the strains studied also showed their abundance significantly varied in EA and HE extracts from strain to strain of *S. novaecaesareae* (Figure 6B,C, Supplementary Table S1). Asp85 produced the highest abundance of 2-(4-bromophenyl)-5-[(4-chlorophenyl) methylsulfanyl]-1,3,4-oxadiazole (EA108) and margaroylglycine (EA77) (Figure 6B). Asp49 extract was characterized by having the highest abundance of [(2R)-2-hydroxy-3-[(9Z,12Z)-nonadeca-9,12-dienoyl] oxypropyl] (5Z,8Z,11Z,14Z)-icosa-5,8,11,14-tetraenoate (EA33) (Figure 6B). Buddlenoid A (HE81) was among the compounds of highest abundance in three of the five isolates studied, Asp85, Asp59 and Asp58 (Figure 6C). N-[2-(cyclohexen-1-yl) ethyl]-3-[2-(cyclohexylcarbamoylamino)-1,3-thiazol-4-yl] propanamide (HE46) was the most abundant compound found in hexane of Asp 49 and Asp58 isolates (Figure 6C). Stearoylethanolamide (HE20), 9 9-[(3-fluoropyridin-4-yl) methyl]-4-pyridin-2-yl-1-oxa-9-azaspiro [5.5]undecan-4-ol (HE9), and (2-hydroxy-3-phosphonooxypropyl) 22-methyltetracosanoate (HE28) was produced in high abundance in the hexane extract of isolate Asp85 (Figure 6C).

### 3.2. PLS-DA Variable Importance Projection (VIP) Scores

**Figure 7:**
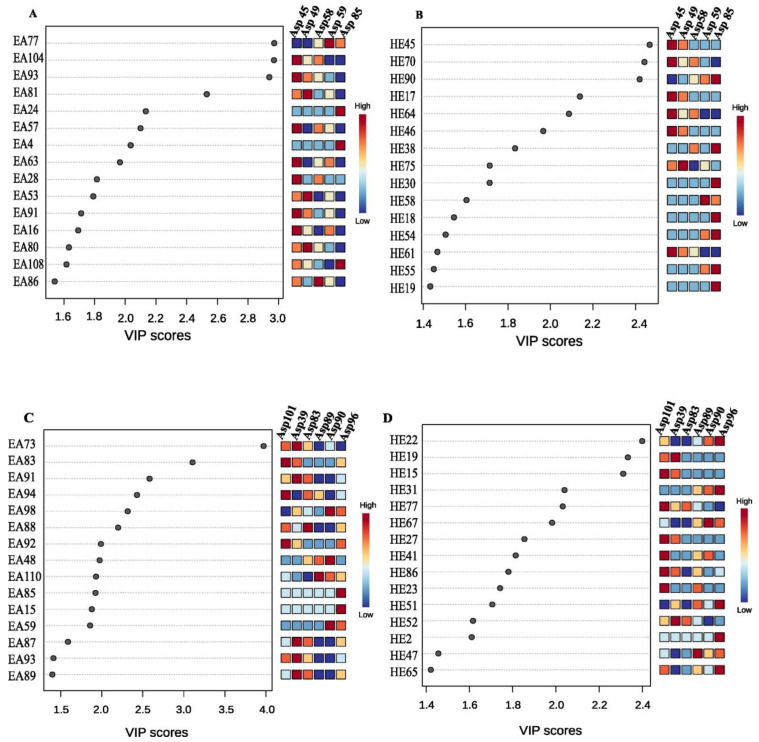
PLS-DA variable importance projection (VIP) scores of metabolites in (**A**,**B**) ethyl acetate (EA) and (**B**,**D**) hexane (HE) extracts of different strains of *S. nojiriensis* (**A**,**B**) and *S. novaecaesareae*. (**C**,**D**). Higher the VIP score of a metabolite, better it is as a biological marker to contribute with the differentiation of treatments. The abundance of metabolites in each sample is shown as a color scale from red (highest abundance) to blue (lowest abundance). Check Supplementary Table S1 for the full identification of compounds represented in this figure.

Several compounds from EA and HE of *S. nojiriensis* and *S. novaecaesareae* were identified as useful biological markers to differentiate isolates from these species due to very high PLS-DA variable importance projection (VIP) scores obtained (Figure 7). Ethyl,5-(4-chloro-2-methoxy-5-methylanilino)quinazoline-2-carboxylate (EA73) appeared to contribute to the separation with the highest score on isolates of *S. nojiriensis* extracted with ethyl acetate. The level of this compound was significant higher in Asp39 (9.6 × 10^14^) and not even produced in Asp89 and Asp96 (Figure 7A). A similar trend was observed in isolates of *S. nojiriensis* extracted with hexane, where 2-[[3-(2-bromophenyl)-1,2,4-oxadiazol-5-yl]methyl]-6-(4-butoxyphenyl)pyridazin-3-one (HE22) had the highest level in Asp96 (7.5 × 10^14^) and were not even produced in Asp39, Asp83 (Figure 7B).

In the case of *S. novaecaesareae,* margaroylglycine (EA77) was the most informative compound for the separation of EA extracts of different isolates of *S. novaecaesareae*, particularly because of its high abundance in Asp59 EA extract (VIP = 3.0; *p =* 9.6 × 10^14^) (Figure 7C). A similar trend was observed for isolates of *S. novaecaesareae* extracted with hexane, with Leu Leu Thr (HE45) showing a high VIP score (VIP = 2.5) to differentiate Asp45 (*p =* 8.7 × 10^14^) and Asp49 (*p =* 7.6 × 10^14^) HE extracts, from Asp58, Asp59, and Asp85 HE extracts, for example (Figure 7D).

### 3.3. Insecticidal Activity

Crude extracts from all investigated *S. novaecesareae* and *S. nojiriensis* isolates caused varying mortality levels in the first instars of *S. frugiperda* (Table 1). The mortality of the first instars of *S. frugiperda* fed on artificial diet treated with ethyl acetate extracts from different isolates of *S. novaecesareae* ranged from 9.7% (IIL-Asp85b) to 66.7% (IIL-Asp59), while hexanic extracts induced from 26.4% (IIL-Asp49) to 100% (IIL-Asp85b) of larval mortality. The mortality of the first instars of *S. frugiperda* fed on artificial diet treated with ethyl acetate extracts from different isolates of *S. nojiriensis* ranged from 6. 9% (IIL-Asp90) to 62.5% (IIL-Asp39), and from 6.9% (IIL-Asp90) to 30.2% (IIL-Asp83) when fed on diets treated with hexanic extracts (Table 1).

### 3.4. Bio-Guided Isolation of Active Compounds

The bio-guided isolation of active compounds after the fractionation of the crude extract of Asp58 (AE58) identified two fractions with moderate insecticidal activity, AE58M and AE58H, against *S. frugiperda* larvae (Table 2).

The fractionation of the crude extract HE59 of the isolate Asp59 yielded 12 fractions that were pooled in six fraction groups by chemical composition similarities, HE59A (1.6% yield), HE59B (37.3%), HE59C (43.6%), HE59D (7.9%), HE59E (0.9%), and HE59F (8.7%). Bio-guided assays against *S. frugiperda* larvae resulted in significative mortality induced by fraction HE59B (82.8 ± 0.2%), when compared to the remaining HE59 fractions (HE59A = 13.9 ± 0.4%; HE59C = 8.6 ± 0.2%; HE59D = 5.4 ± 0.5%; HE59E = 6.8 ± 0.6%; H59F = 5.9 ± 0.4%) (GLM with quasi-binomial distribution; *F =* 2.34; *p* < 0.001). The fractionation of fraction HE59B using an LC preparative system yielded 48 fractions, which were pooled into five groups: A: subfractions 1–12, B: 13–22, C: 23–32, D: 33–37, and E: 38 to 48. The bioassay with these group fractions demonstrated the insecticidal activity of the crude extract HE59 was predominantly represented in group D (33–37 subfractions), which caused 82.8% mortality of the first instars of *S. frugiperda* (Table 2).

### 3.5. Reference Lethal Concentrations

The estimated concentrations of the H59B fraction required to cause 50 (LC_50_) and 90% (LC_90_) mortality of *S. frugiperda* population by ingestion were 10.821 and 26.254 µg/µL, respectively, while for the AE58M fraction, estimated LC_50_ and LC_90_ values were 15.051 and 38.845 µg/µL, respectively (Table 3).

### 3.6. Identification of Insecticidal Compounds from Asp58 and Asp59

**Figure 8:**
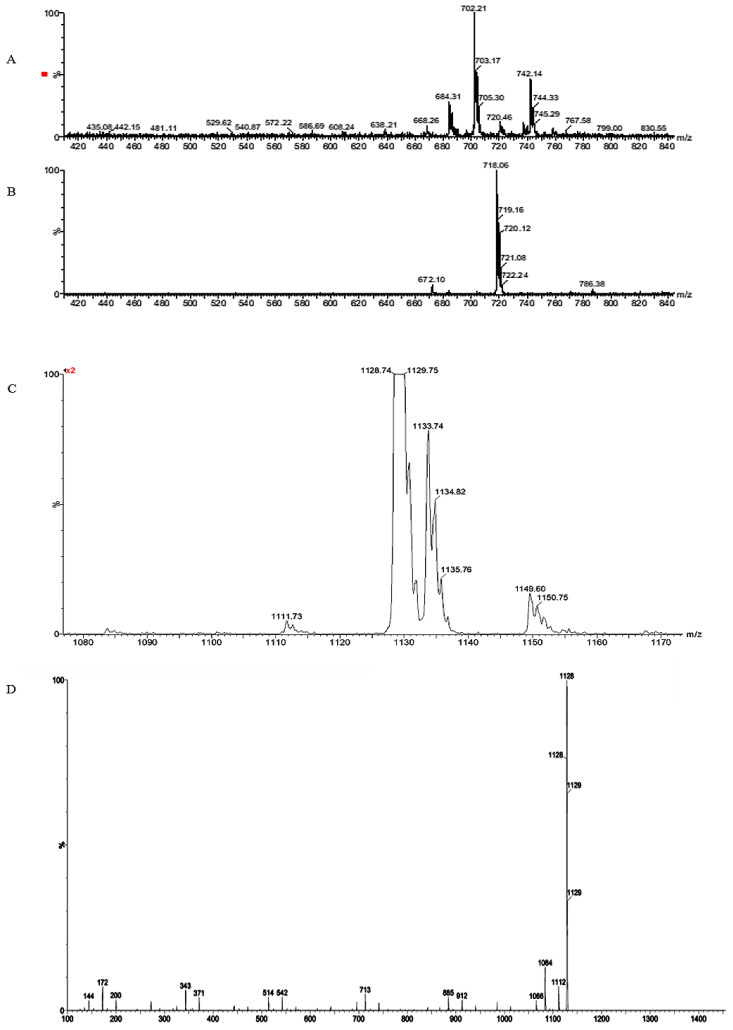
ESI+ (**A**) and ESI− (**B**) spectra of the active fraction AE58M of the ethyl acetate crude extract from the isolate Asp58 of *S. novaecaesareae*; (**C**) MS-ESI+ spectrum of the active fraction HE59B-D from the isolate Asp59 of *S. nojiriensis* and (**D**) CID spectrum of ion 1128 *m*/*z*.

An LC-MS analysis of the active fraction of the ethyl acetate crude extract isolate IILab-Asp58 led to the identification of compounds of the molecular mass of 718 u. In ESI+ were observed the ions of *m*/*z* 742 and 702, which were assigned to [M + Na]^+^ and [M + H-H_2_O]^+^, respectively (Figure 8A,B). In ESI negative was observed the ion of *m*/*z* 718. In all peaks were observed the natural abundance isotope ratio of ^35^Cl and ^37^Cl, indicating the presence of a chlorine group in the structure. A search in the Dictionary of Natural Products database for mass, biological source, and chlorine led to the identification of the active compound in the fraction as naphtomycin A (Figure 8) [47].

An LC-MS analysis of the active fraction of Asp59 showed another compound in a cluster of ion adducts with H^+^ (*m*/*z* 1111), Na^+^ (*m*/*z* 1133), NH_4_^+^ (*m*/*z* 1128), and K^+^ (*m*/*z* 1149) (Figure 8C), indicating the active compound has a molecular mass of 1110. The CID experiment to the ion of *m*/*z* 1128 showed successive neutral losses of 99, 72, and 100 u, which were assigned to losses corresponding to the valine [Val-H_2_O], acid [Lac-H_2_O], and 2-hydroxisovaleric acid [Hiv-H_2_O], respectively. A DNP database search putatively identified the bioactive compound as valinomycin, and this was corroborated by the identification from an LC-MS/MS analysis (Figure 8C,D) [48].

## 4. Discussion

Our additional data on the putative identification of the chemical diversity produced by different isolates of *S. nojiriensis* and *S. novaecaesareae* support the previous information on the intraspecific chemometric diversity of these *Streptomyces* species [44]. Our data also support the challenge of the “one strain many compounds” (OSMAC) approach for the exploitation of the full metabolic potential of microorganisms of interest through the utilization of different cultivation methods [44,49], as very high chemical diversity was identified among isolates belonging to *S. nojiriensis* and to *S. novaeceasareae* under identical culture conditions. Moreover, the isolates of *S. novaecesareae* produced different bioactive insecticidal molecules against the first instars of *Spodoptera frugiperda.* The intraspecies variation we reported for the ant-associated symbiont *S. novaecesareae* has also been recently reported for several actinobacteria, including *Streptomyces* species isolated from different environments [42,50,51]. Moreover, our data demonstrate that intraspecies variation can occur within a single niche as opposed to the arguments that the intraspecies variation in antibiotic production in *Streptomyces* is an adaptative trait evolved due to the need to outcompete distinct bacteria from different niches [42].

The mechanisms underlying the intraspecific chemical diversities and the diverse biological activity exhibited especially by *S. novaecesareae*, particularly when derived from a single source and subjected to identical nutritional and environmental conditions, remain a subject of limited understanding.

The cyclodepsipeptide ionophore valinomycin we identified causing insect mortality in extracts from *S. novaecesareae* Asp59 is a common metabolite produced by several *Streptomyces* strains. The valinomycin biosynthetic gene cluster consists of two large nonribosomal synthetase genes that lead to valinomycin production in *Streptomyces* [52]. The occurrence of differences in the number of biosynthetic gene clusters (BGCs) has been shown as an explanation for the intraspecific metabolic variation reported in *Streptomyces*. Genomic analyses of *S. hygroscopicus* collected from different sites displayed a great variation in BGCs [53], but significant differences in BGCs were also identified in two co-isolated strains of *Streptomyces* sp., with only one of them being capable to synthesize staurosporine [54]. Such variations in BGCs may occur due to horizontal gene transfer (HGT) [55,56], but broad evolutionary analyses have demonstrated that only one gene has been acquired at every 100,000 years by *Streptomyces* lineages disfavoring HGT as a common event of BGCs [57]. Analyses of the valinomycin gene clusters in *Streptomyces* identified that valinomycin-producing strains form a well-defined phylogenetic group, providing strong support for the vertical transmission of valinomycin gene cluster in *Streptomyces* [58], giving little support to HGT to explain the intraspecies variation observed in *S. novacaesareae*. It is more likely the intraspecies variation observed in the production of valinomycin occurs due to mutations that lead to the loss of function of genes belonging to valinomycin BGCs [58,59].

Valinomycin has been shown to be produced in all strains that carry this BGC, indicating this compound confers adaptive advantages to valinomycin producers, even though negative effects were not observed in valinomycin mutants under laboratory conditions [52,59]. The ionophore valinomycin carries both hydrophobic and hydrophilic moieties that enable it to effectively transport ions across lipid-based membrane barriers of living cells, and it has been reported to exhibit a wide range of bioactivities, including antitumor, antibacterial, antibabesia, and antifungal effects [60,61,62,63], including insecticidal activity [64]. Nonetheless, the exact ecological role it plays to valinomycin producers remains unknown.

Valinomycin was not detected in the remaining isolates of *S. novaecaesareae,* but isolates Asp58 and Asp85b were found to produce nonpolar, highly active insecticidal compounds. The insecticidal compound produced by Asp85b was not identified, but the insecticidal activity produced by Asp58 was identified as naphthomycin A. Naphthomycin was shown to be assembled using 3-amino-5-hydroxybenzoic acid (AHBA) as a starter unit that uses a cluster of six genes. Two acyl ACP ligase are available and thought to load AHBA onto the polyketide synthase. The BGC still carry two additional genes involved in naphthomycin ring closure (*napB*) and naphthomycin modification (*napA1*) [65]. There is no evidence from the literature that the naphthomycin gene cluster is maintained in *Streptomyces* by vertical transmission as the valinomycin gene cluster. Naphthomycin synthesis by isolate Asp58 could then represent an event of recombination intra and/interspecies, as the genomic analysis of streptomycetes has proven they carry high levels of recombination [56,57,66,67]. Naphthomycin is a naphthoquinone derivative known to be produced by other species of *Streptomyces*. Naphthomycin has been demonstrated to be a natural compound with bactericide [47], fungicide [68], and antineoplastic activities [69]. This is the first report of naphthomycin as an insecticidal compound.

Actinobacteria is a well-known source of bioactive molecules, and *Streptomyces* is by far the best-investigated genus of actinobacteria, with an infinite list of natural compounds identified for a range of purposes [70,71]. But bioactive molecules with insecticidal activity have been reported for several other actinobacteria genera [44,72,73]. The most important natural insecticides identified from Actinobacteria were identified as spinosyns in the fermentation products of *Saccharopolyspora spinosa* and *S. pogona* [74,75]. We did not detect spinosyns or spinosoids in the crude extracts of isolates with insecticidal activity, but strong insecticidal activity was identified for naphtomycin A and valinomycin from extracts of Asp58 and Asp59, respectively.

Valinomycin and naphthomycin A are natural products that have shown promising insecticidal activity, making them potential candidates for environmentally friendly pest control strategies. Additionally, chemical insecticides typically target specific pathways in insect physiology, potentially leading to resistance development [76]. In contrast, the complex structures of valinomycin and naphthomycin could make them less persistent in the environment, due to the high volatility, providing a potential advantage in sustainable pest management.

Valinomycin and naphtomycin by *Streptomyces* species are promising natural insecticides due to their unique modes of action—disrupting potassium ion gradients and mitochondrial function. These compounds are potentially environmentally friendly, likely with low toxicity towards non-target organisms and a reduced risk of bioaccumulation [77]. However, challenges include potential toxicity to non-target species, the possibility of resistance evolution, and the complexities and costs associated with large-scale production [78,79]. Regulatory hurdles and the need for thorough safety evaluations may also pose significant barriers for their commercialization. Despite these challenges, their inclusion in integrated pest management (IPM) programs could reduce the reliance on synthetic pesticides, aligning with the increasing demand for sustainable agricultural practices [80]. In conclusion, the present study demonstrated that different isolates of *S. novaecaesareae* and *S. nojiriensis* exhibit substantial metabolic diversity, with the synthesis of a range of different compounds among intraspecies isolates. The intraspecies chemical diversity observed and the potential biological activity of the different compounds synthesized hold great promise for their bioprospection. Our data demonstrate different approaches will have to be employed for the full exploitation of biotechnological potential of insect-associated symbionts.

## Figures and Tables

**Table 1:** Larval mortality (%) of *Spodoptera frugiperda* when fed on surface-treated artificial diets with crude extracts (264 µg/cm^3^) of isolates of *S. nojiriensis* and *S. novaecaesareae*, after 120 h of exposure under controlled laboratory conditions (25 ± 1 °C; 60 ± 10% RH; 14 h photophase).

Phylotype		Mortality % (±SE)		
	IILab Code	Control	Ethyl Acetate	Hexane
*S. nojiriensis*	IILab Asp101	0	22.2 ± 0.2	8.0 ± 0.3
	IILab Asp89		11.7 ± 0.5	11.8 ± 0.3
	IILab Asp90		6.9 ± 0.3	6.9 ± 0.5
	IILab Asp83		15.3 ± 0.4	30.2 ± 0.7
	IILab Asp96		12.7 ± 0.4	10.3 ± 0.3
	IILab Asp39		62.5 ± 0.3	12.0 ± 0.3
*S. novaecaesareae*	IILab Asp45	0	20.8 ± 0.5	27.8 ± 0.5
	IILab Asp49		65.3 ± 0.4	26.4 ± 0.4
	IILab Asp58		61.1 ± 0.5	29.2 ± 0.5
	IILab Asp59		66.7 ± 0.5	91.6 ± 0.3
	IILab Asp85		9.7 ± 0.3	100 ± 0

**Table 2:** Lethal effects (mean ± SE) of the ethyl acetate extract fractions of isolate Asp58 (AE58) and hexane extract fractions of isolate Asp59 (HE59) (tested according to their equivalent concentrations) on *S. frugiperda* by the ingestion route (25 ± 1 °C; 60 ± 10% RH; 14 h photophase).

Isolate	Fraction	Mortality (%) (±SE) ^1^
Asp 59	HE59B-A	15.8 ± 0.6 b
	HE59B-B	15.9 ± 0.4 b
	HE59B-C	18.4 ± 0.5 b
	HE59B-D	82.8 ± 0.2 a
	HE59B-E	3.8 ± 0.4 b
	Control	5.0 ± 0.2 b
	*p*-value	<0.001
	*F*	3.503
Asp 58	AE58H	29.8 ± 0.2 a
	AE58M	31.6 ± 0.2 a
	Control	5.0 ± 0.2 b
	*p*-value	<0.001
	*F*	15.85

^1^ Means followed by different letters within the column indicate significant differences between treatments using a GLM with quasi-binomial distribution followed by Tukey’s post hoc test (*p*< 0.05).

**Table 3:** Estimated LC_50_ and LC_90_ at µg/µL of the selected crude extracts on neonates of *S. frugiperda* by the ingestion exposure route after 120 h (25 ± 1 °C; 60 ± 10% RH; 14 h photophase).

Treatment	n ^1^	Slope ± SE ^2^ (*p* Value)	LC_50_ (CI) ^3^	LC_90_ (CI) ^3^	χ^2 4^	*df* ^5^
HE59B	576	3.329 ± 0.259 (*p* < 0.001)	10.821 (9.996–11.715)	26.254 (22.452–30.6222)	3.622	6
AE58M	576	3.112 ± 0.245 (*p* < 0.001)	15.051 (13.808–16.406)	38.845 (32.475–46.464)	6.012	6

^1^ n: number of tested insects; ^2^ SE: standard error; ^3^ CI: 95% confidence interval; ^4^ χ^2^: calculated chi-square value; ^5^ *df*: degrees of freedom.

## Data Availability

The datasets generated in the current study are available from the corresponding author on reasonable request.

## Supplementary Materials

The following supporting information can be downloaded at: https://www.mdpi.com/article/10.3390/insects15090707/s1, Table S1: Mass spectrometry data and putative identification of all metabolites produced by the *S. nojiriensis* and *S. novaecaesareae* isolates studied using ethyl acetate and acetone extracts.
